# Evaluation of the Potential of Novel Co-Processed Excipients to Enable Direct Compression and Modified Release of Ibuprofen

**DOI:** 10.3390/pharmaceutics16111473

**Published:** 2024-11-19

**Authors:** Ivana Aleksić, Teodora Glišić, Slobodanka Ćirin-Varađan, Mihal Djuris, Jelena Djuris, Jelena Parojčić

**Affiliations:** 1 Department of Pharmaceutical Technology and Cosmetology, Faculty of Pharmacy, University of Belgrade, Vojvode Stepe 450, 11221 Belgrade, Serbia; teodora.glisic@pharmacy.bg.ac.rs (T.G.); slobodanka.cirin-varadjan@outlook.com (S.Ć.-V.); jelena.djuris@pharmacy.bg.ac.rs (J.D.); jelena.parojcic@pharmacy.bg.ac.rs (J.P.); 2 Department of Catalysis and Chemical Engineering, Institute of Chemistry, Technology and Metallurgy—National Institute of the Republic of Serbia, University of Belgrade, Njegoševa 12, 11000 Belgrade, Serbia; mihal.djuris@ihtm.bg.ac.rs

**Keywords:** lipid excipients, detachment stress, ejection stress, sticking, prolonged release, fluidized bed melt granulation, co-processing, compaction analysis, glyceryl palmitostearate, lactose monohydrate

## Abstract

**Background/Objectives**: Improving the production rates of modern tablet presses places ever greater demands on the performance of excipients. Although co-processing has emerged as a promising solution, there is still a lack of directly compressible excipients for modified-release formulations. The aim of the present study was to address this issue by investigating the potential of novel co-processed excipients for the manufacture of modified-release tablets containing ibuprofen. **Methods**: The excipients were prepared by melt granulation of lactose monohydrate with glyceryl palmitostearate as a binder. The influence of glyceryl palmitostearate particle size, ibuprofen content, compression pressure, and compression speed on the compaction behavior of the tablet blends was analyzed. **Results**: Novel co-processed excipients ensured good flowability and acceptable mechanical properties of the tablets containing up to 70% ibuprofen. Furthermore, lipid-based co-processed excipients proved to be very promising for directly compressible formulations with high-dose, highly adhesive active pharmaceutical ingredients such as ibuprofen, as they do not require additional lubricants. The influence of compression speed on the tensile strength of the tablets prepared was not pronounced, indicating the robustness of these directly compressible excipients. The investigated lipid-based excipients enabled a prolonged release of ibuprofen over 10 h. **Conclusions**: The novel lipid-based co-processed excipients have shown great potential for directly compressible formulations with modified release of high-dose, challenging active pharmaceutical ingredients.

## 1. Introduction

Although tablets can be prepared by various methods, they are most commonly prepared by compression of powder particles or granules [1]. Compression of the mixture of the active pharmaceutical ingredient (API) and excipients usually requires granulation prior to compression into tablets due to poor flowability and/or compression properties. The use of directly compressible diluents can in some cases help to overcome the problems associated with the poor flow/compaction properties of the API and enable the use of direct compression as the simplest, most time, energy, and cost-efficient method of tablet manufacturing. For this reason, great efforts have been made to improve the functionality of commonly used excipients, such as lactose, by modifying the particle size, morphology, crystallinity, porosity, and surface area through the application of various processing techniques [2]. However, the requirements for good flowability and good compression behavior often pose conflicting demands on particle engineering. Therefore, it can be a great challenge to modify a single excipient to achieve improved flowability and good compaction properties while maintaining high dilution capacity, all of which are required for direct compression.

The increasing demand for high-performance excipients is being further driven by innovations and improvements in tablet presses. Modern tableting machines can produce hundreds of thousands of tablets per hour up to more than one million tablets per hour [3,4,5]. It has been reported that various tableting issues, such as die wall and punch-sticking, tablet defects (e.g., capping and lamination), and weight variations, are becoming more prominent in high-speed production [6,7,8]. With the rapid improvement in production rates of modern tableting machines, the requirements for directly compressible formulations are constantly increasing. Furthermore, compression per se is a continuous process. The development of equipment that enables the accompanying unit operations in direct compression (e.g., weighing and mixing) to be carried out continuously, as opposed to traditional batch processing, and the introduction of process analytical technology (PAT) as a tool for process control and monitoring, have brought direct compression even more to the fore as the method of choice in tablet production. However, this places even higher demands on the performance of directly compressible excipients, especially for formulations with APIs characterized by poor flow and/or compression properties [9]. The development of co-processed excipients has arisen as a promising solution to these growing challenges. The International Pharmaceutical Excipients Council defines a co-processed excipient as a combination of two or more excipients designed to physically alter their properties in a way that cannot be achieved by simple physical mixing, and without significant chemical alteration [10]. An appropriate processing technique is applied to achieve a purely physical interaction between excipients, leading to improved functionality and synergy between them [11]. Conventional methods such as spray drying and wet granulation are still most commonly used for co-processing [2,11,12]. However, a few recent studies have shown the great potential of melt granulation as a more environmentally friendly method for the production of high-performance, multifunctional co-processed excipients [13,14,15,16].

Since the introduction of co-processing in the late 1980s, various co-processed excipients have been developed for direct compression of immediate-release tablets, many of which are intended for orally disintegrating tablets [17,18]. Many of these excipients are lactose-based [2,19]. Interestingly, there is only one commercially available co-processed excipient that is designed for direct compression of modified-release formulations, namely, lactose co-processed with hypromellose [20,21]. There are few reports in the scientific literature on the development of directly compressible, co-processed excipients for modified-release formulations, and these generally involve complex and/or energy-intensive preparation methods [22,23]. For example, Patel and coworkers prepared the co-processed excipient consisting of glyceryl monostearate, dicalcium phosphate dihydrate, and polyvinylpyrrolidone K30 by wet granulation [22]. Serrano-Mora et al. developed a co-processed excipient for controlled-release formulations by preparing solid lipid nanoparticles of Compritol^®^ 888 ATO and adsorbing them onto a directly compressible dicalcium phosphate dihydrate [23].

Despite the tremendous efforts that have been directed towards the development of directly compressible excipients in recent decades, formulations with a high API content represent a major challenge. Highly dosed APIs greatly affect the overall processability of the tableting mixture, making wet granulation often the only choice [24,25]. Ibuprofen is a non-steroidal anti-inflammatory drug (NSAID) that is widely used in solid oral dosage forms. It is administered in relatively high single therapeutic doses, ranging from 200 to 800 mg [26]. Its poor flowability, poor compression properties, and a strong tendency to stick to punch surfaces during tablet compression are well-known and widely described in the literature. These properties in combination with the high ibuprofen content in the tableting mixture make the tableting of ibuprofen formulations quite challenging and in most cases lead to a granulation step prior to tableting [27,28,29,30].

Given these challenging properties, ibuprofen was selected in this study as a model API for direct compression with novel co-processed excipients containing lactose monohydrate and Precirol^®^ ATO5 (glyceryl palmitostearate). In our previous studies, lipid-based co-processed excipients have shown great potential for direct compression, not only in terms of their good flowability and compactability but also their antiadhesive and lubricating properties [15,16]. In addition, lipid excipients are known as matrix-forming agents in modified-release tablets [31,32]. However, to our knowledge, lipid excipients have not yet been used to prepare co-processed lactose-based excipients for modified-release formulations. This article is a revised and expanded version of a paper entitled ‘From co-processing by melt granulation towards direct compression of high ibuprofen loaded formulations’, which was presented at the 14th CESPT, Ohrid, North Macedonia, 28–30 September 2023. Namely, the present study builds on the results of the conference paper on the influence of formulation and compression-related parameters on the compaction behavior of novel co-processed excipients [33]. This research was continued and the potential of co-processed excipients for use in formulations with prolonged release was evaluated. Therefore, the aim of the present study was to evaluate the suitability of novel co-processed excipients obtained by in situ fluidized bed melt granulation for the production of modified-release tablets with challenging, high-dose API by direct compression. More specifically, the goal of this study was to investigate the influence of initial particle size of Precirol^®^ ATO5, ibuprofen content, compression pressure, and compression speed on the compaction behavior of ibuprofen tablet blends as well as ibuprofen dissolution from directly compressed tablets.

## 2. Materials and Methods

### 2.1. Materials

Co-processed excipients were prepared by using glyceryl palmitostearate (Precirol^®^ ATO 5 Gattefossé S.A.S, Saint-Priest Cedex, France) as a meltable binder and lactose monohydrate (Carlo Erba Reagents, Milan, Italy) as a filler. Ibuprofen (Fagron, Rotterdam, The Netherlands) was selected as the model drug. Sodium Hydroxide (Fisher Scientific, Loughborough, UK), potassium phosphate monobasic (Sigma-Aldrich Chemie GmbH, Steinheim, Germany), and hydrochloric acid (Avantor Performance Materials Poland S.A., Gliwice, Poland) were used for dissolution media preparation.

### 2.2. Characterization of the Model Drug

The particle size of ibuprofen was analyzed using the Mastersizer 3000 laser diffraction particle size analysis system with the Aero S accessory (Malvern Instruments Ltd., Worcestershire, UK). The analysis was performed with a 2% laser obscuration ratio, at a feed rate of 50% and an airflow of 1 bar. The measurement was repeated 4 times, and the results were displayed as the average of d10, d50, d90, and Span.

### 2.3. Co-Processing by Melt Granulation

Co-processing by in situ melt granulation was carried out in a Mycrolab fluid bed processor (OYSTAR Hüttlin, Schopfheim, Germany) at a batch size of 200 g. Lactose monohydrate (85%) was granulated with Precirol^®^ particles (15%) from different size fractions, i.e., sieve fractions of 125–180 μm (≈150 µm) or 600–710 μm (≈655 µm). The powders were fluidized and the point at which the product temperature reached 65 °C was considered the starting point of granulation. During granulation, the product temperature was maintained at this temperature and the inlet air flow rate was approximately 30 m^3^/h. At 10 min after the start of granulation, the inlet air heating was switched off, and when the product temperature dropped below 30 °C, the fluid bed processor was stopped.

### 2.4. Co-Processed Excipients’ Particle Size and Shape Analysis

The shape and the size of the particles were assessed by 2D image analysis. The images were taken with HP Scanjet 300 scanner (Hewlett Packard Enterprise, Houston, TX, USA) at a scanning resolution of 4800 dpi. The open-source software ImageJ 1.53m was used for image processing, and the aspect ratio (AR), circularity (C), and roundness (R) were calculated for particle shape estimation [34].

The aspect ratio is determined as the ratio of the major and minor particle axis/diameter.

The circularity is calculated by the following equation:

(1) $$C=4\pi \frac{A}{P^{2}}$$

where A is the projected area of a particle, while P is the perimeter of a two-dimensional particle outline.

The roundness is calculated by the following equation:

(2) $$R=4\frac{A}{\pi d_{m}^{2}}$$

where A is the projected area of a particle, while d_m_ is the major axis of a particle.

The particle size was evaluated by projected diameter (d_A_) that is calculated by the following equation:

(3) $$d_{A}=\sqrt{4\frac{A}{\pi }}$$

where A is the projected area of a particle.

### 2.5. Carr Index Determination

The bulk density and the tapped density (1250 taps) of the ibuprofen, the co-processed excipients, and their mixtures with ibuprofen were determined using the graduated cylinder and the volumeter STAV 2003 (J. Engelsmann AG, Ludwigshafen, Germany). The measurements were performed in triplicate and the results obtained were used to calculate the Carr index (CI) by the following equation:

(4) $$CI=100\times \frac{\rho_{t}-\rho_{b}}{\rho_{t}}$$

where ρ_t_ is the tapped density and ρ_b_ is bulk density.

### 2.6. Experimental Design

Tableting mixtures containing novel co-processed excipients and ibuprofen were prepared and characterized according to a 2^4^ full factorial design (Table 1). The mixtures were prepared by mixing the appropriate amount of ibuprofen and the co-processed excipient in a powder mixer (Farmalabor, Milan, Italy) at 50 rpm for 10 min. The effect of binder particle size, ibuprofen content, compression load, and compression speed on compaction properties was investigated. The experiments were conducted in randomized order and the results were evaluated by analysis of variance, at the 0.05 level of significance, using Design-Expert^®^ software (version 7.0, Stat-Ease Inc., Minneapolis, MN, USA).

### 2.7. Tablet Compression and Compaction Analysis

The tablets were compressed on an instrumented single-punch tablet press (GTP D-series, Gamlen Tableting Ltd., Nottingham, UK) with 6 mm flat-faced punches. The compact mass was 100 mg. Force–displacement curves were generated during compaction, and were subsequently used for compaction analysis and calculation of the following parameters: network of compression (NW), detachment stress (DS), and ejection stress (ES).

The network of compression was calculated as the difference between the area under the force–displacement curve during the compression phase (corresponding to the total work of compression) and during the decompression phase (corresponding to the work of elastic deformation). The area under the curve was calculated according to the trapezoidal rule.

The detachment stress and the ejection stress were calculated using the following equations:

(5) $$DS=\frac{F_{d}}{r^{2}\pi }$$

(6) $$ES=\frac{F_{e}}{\pi dt}$$

where F_d_ is the maximum force recorded during the detachment phase, F_e_ is the maximum force recorded during the ejection phase, r is the compact radius, d is the compact diameter, and t is the compact thickness.

Elastic recovery was calculated using the following equation [35]:

(7) $$ER=\frac{t_{1}-t_{0}}{t_{0}}\times 100$$

where t_0_ is the compact thickness under the maximum compression pressure inside the die, while t_1_ represents compact thickness 24 h after compression outside the die.

At least three compacts were prepared for each experimental setup and the results were presented as the mean value.

### 2.8. Tensile Strength

Diameter of the compact and compact resistance to crushing were determined using the Erweka TBH 125D tablet hardness tester (Erweka GmbH, Langen, Germany). Compact thickness was measured using a caliper, and all measurements were performed 24 h after compression. The obtained results were used to calculate tensile strength according to the following equation:

(8) $$\sigma =\frac{2\cdot F}{\pi \cdot d\cdot t}$$

where F is the compact crushing force, d is the compact diameter, and t is the compact thickness.

### 2.9. Strain Rate Sensitivity

Properties of the compacts compressed at different compression speeds were compared by strain rate sensitivity (SRS) calculated by the following equation [36]:

(9) $$SRS=\frac{\left|\sigma_{2}-\sigma_{1}\right|}{\sigma_{1}}\times 100$$

where σ_1_ represents compact tensile strength at low compression speed, and σ_2_ is compact tensile strength at high compression speed.

### 2.10. Dissolution Test

The dissolution of ibuprofen from the investigated tablets (prepared at a compression pressure of 500 kg and a compression speed of 60 mm/min) was tested in the reciprocating cylinder apparatus (VanKel Technology Group, Cary, NC, USA) using the media change method as follows: 0.1 M HCl (2 h) and phosphate buffer pH 6.8 (6 h, with a vessel change after 2 h). In each vessel, volume of the dissolution medium was 250 mL, mesh size was 405 μm both at the top and at the bottom of the cylinder, and apparatus was operating at 10 dpm. The experiments were run in triplicate, and dissolution media temperature was maintained at 37 ± 0.5 °C. Withdrawn dissolution media samples were assayed UV spectrophotometrically (Evolution 300 spectrophotometer, Thermo Fisher Scientific, Madison, WI, USA) at 221 nm.

### 2.11. Analysis of Drug Release Kinetics

The kinetics of the release of ibuprofen from the prepared tablets was analyzed by fitting the dissolution test results to common mathematical models, including zero-order, first-order, Higuchi, and Korsmeyer–Peppas models.

## 3. Results and Discussion

### 3.1. Properties of Ibuprofen and Co-Processed Excipients

Ibuprofen is described in the literature as needle-shaped crystals with a rough surface, which exhibit strong cohesive and adhesive properties and are therefore poorly flowable [30,37]. In accordance with the literature, the results of the present study showed poor flow properties of ibuprofen, as indicated by the relatively high value of the Carr index, which corresponds to poor flow behavior according to the European Pharmacopeia (Table 2). The pronounced adhesiveness and cohesiveness were also visually apparent when handling the powder and testing the bulk and tapped density.

Three parameters were used to evaluate the shape of the particles of co-processed excipients: the aspect ratio, the circularity, and the roundness. For ideally spherical particles, all parameters would be equal to one. While the value of AR indicates the elongation of the particles, the value of C can be influenced by the roughness of the particles [38].

**Figure 1:**
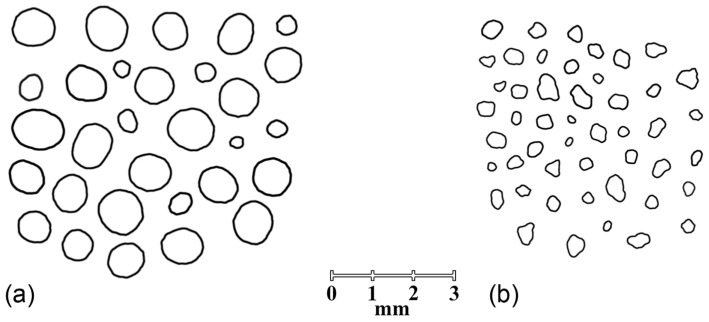
The contours of the particles of two co-processed excipients: (**a**) C655 and (**b**) C150.

The values of the shape parameters determined for the two co-processed excipients are listed in Table 3. All three shape parameters indicate a more spherical shape of the particles of the co-processed excipients obtained with a larger initial Precirol^®^ particle size (C655) compared to the particles obtained by co-processing lactose monohydrate with a binder of smaller particle size (C150). It was reported that the shape of the granules obtained by fluidized bed melt granulation can be influenced by the agglomeration mechanism involved. Immersion and layering as an agglomeration mechanism, which is predominant when larger-sized binder particles are used, usually lead to highly spherical granules [32,39]. This could explain the observed differences between the two excipients, i.e., the larger, more spherical, and smoother granules of C655 (Figure 1). Both co-processed excipients showed excellent flowability, as shown in Table 3 [1]. The lower value of CI for C655 can be attributed to the observed differences in particle size and morphology of the two excipients.

### 3.2. Flow Properties

**Figure 2:**
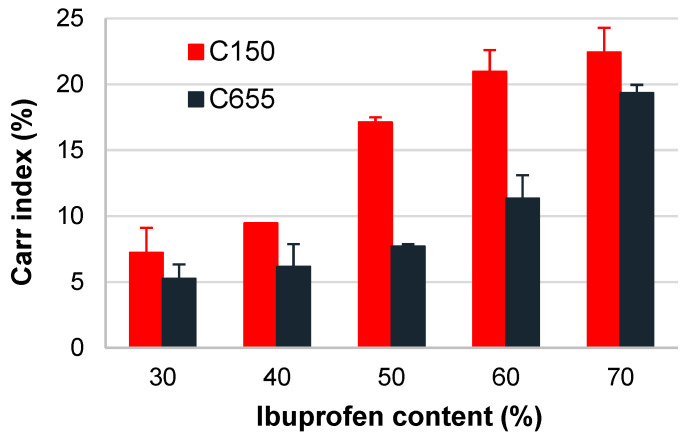
Flowability of the mixtures of co-processed excipients and ibuprofen.

Figure 2 shows the flow properties of the mixtures of co-processed excipients with different ibuprofen contents. Depending on the ibuprofen content, the tested samples showed excellent-to-passable flow [1]. For both co-processed excipients, an increase in the Carr index values was observed with increasing content of this poorly flowable, cohesive API. However, even at the highest ibuprofen content tested (70%), both co-processed excipients provided acceptable flowability of the mixtures, i.e., fair flowability in the case of C655 and passable flowability in the case of C150. The flowability of the mixtures with 70% ibuprofen was comparable to the literature results obtained for directly compressible formulations with the same ibuprofen content but with the commercially available co-processed excipient and additional fumed silica as a glidant [29]. Regardless of the ibuprofen content, the mixtures with C655 showed notably better flow properties than those with C150, which, as mentioned above, can be attributed to the differences in particle size and morphology of the two co-processed excipients.

### 3.3. Compaction Behavior

Although tablet production by powder compression is considered a relatively simple, time- and cost-efficient process, it can be very challenging due to the complex interplay of material properties, compression parameters, and the characteristics of the tablet press and tooling used, which can lead to various tablet defects (e.g., capping and lamination, powder sticking to punches and die walls, insufficient mechanical strength, etc.). Therefore, appropriate methods should be used to thoroughly characterize the compaction properties of pharmaceutical materials in the research and development phase. Compaction simulators have been recognized as useful pieces of equipment for tablet compression characterization [36]. Dynamic compaction analysis has been shown to contribute to a better understanding of the compaction behavior of powders and the factors that influence it [15,24,40,41,42].

In this study, dynamic compaction analysis was applied to evaluate the influence of the initial particle size of Precirol^®^ ATO5, ibuprofen content, compression pressure, and compression speed on the compaction behavior of the mixtures of ibuprofen and co-processed excipients. The following parameters were calculated from the force-displacement curves generated during compaction: network of compression (NW), detachment stress (DS), and ejection stress (ES).

NW is used as a measure of the compressibility of powders; i.e., larger NW corresponds to materials that are more susceptible to plastic deformation, with or without fragmentation [43]. The NW of the investigated samples ranged from 0.40 to 0.67 J (Table 4) and was significantly affected by all investigated variables and their two-factor interactions (Table 5). The compression parameters showed the most significant influence on NW with higher NW values at higher compression load and faster compression. The influence of ibuprofen content on NW was more pronounced in the case of C150, with lower NW at higher content of this poorly compressible API in the mixture.

**Figure 3:**
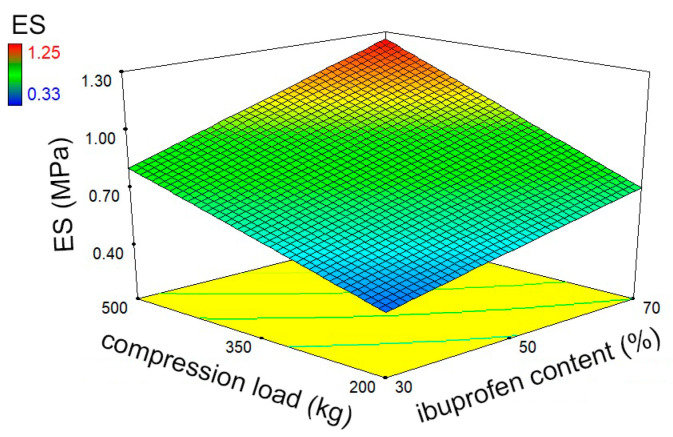
Three-dimensional surface plots showing the effects of ibuprofen content and compression load on ejection stress (at a binder particle size of 655 µm and a compression speed of 120 mm/min).

Two parameters, detachment and ejection stress, were used to evaluate the potential of the novel co-processed excipients to overcome the common problems associated with the high sticking tendency of ibuprofen formulations during compaction. The detachment stress is derived from the detachment force (or take-off force) required to detach the tablet from the surface of the lower punch tip, while the ejection stress is calculated from the ejection force required to eject the tablet out of the die. High values of these parameters are associated with poor lubrication and possible picking and sticking problems. The results obtained showed good lubrication of the investigated samples with relatively low values for both parameters (Table 4). DS was significantly affected by the ibuprofen content and the compression parameters, with all these factors showing a positive effect on the values of DS. The same factors showed a significant influence on ES. The highest value of this response parameter (1.25 MPa) was obtained for the tablets containing 70% ibuprofen, which were prepared at a higher compression load and speed (Figure 3). However, it is still much lower than the highest acceptable value of 5 MPa [44]. Therefore, adhesion problems and related tablet defects (e.g., capping and lamination) are not expected even at 70% ibuprofen, regardless of compression pressure or speed. These results are very promising considering that ibuprofen has been reported in the literature to have a strong tendency to adhere to tablet tooling due to the rough and adhesive surface of its crystals and its low melting point [27,29]. Furthermore, most of the commercially available co-processed excipients do not contain lubricants and thus require their addition. The importance of the addition of common lubricants, such as magnesium stearate and sodium stearyl fumarate, and their effects on the properties of directly compressed tablets prepared with co-processed excipients have been reported in the literature [41,45,46]. Co-processed excipients based on lactose and lipophilic glycerides have already been shown to have superior lubricating properties compared to commercially available co-processed lactose-based excipients, including hypromellose-based excipients for modified-release formulations (Retalac^®^). Namely, in our previous work, we have shown that lipid-based co-processed excipients have significantly lower detachment and ejection stresses compared to this commercially available modified-release co-processed excipient [16]. Milanovic et al. showed that hot-melt coating with Precirol^®^ ATO5 leads to good lubrication of the granules and that no lubricant is required regardless of the applied compression pressure [47]. Vaingankar and Amin reported that the production of granules by hot melt extrusion with the addition of a lipid excipient eliminates the need for conventional lubricants, even at high drug content [48]. This supports the results of our study, which show that lipid-based co-processed excipients can be used for direct compression with highly adhesive APIs such as ibuprofen without additional lubricants.

The obtained elastic recovery values are presented in Table 4. Compression load showed the most significant influence on ER, with higher compression pressure leading to higher values of ER (Table 5). Compression speed also showed a significant effect on ER, which was more pronounced at higher ibuprofen content. Higher ER values were observed when compression was performed faster. Ibuprofen is known for its elastic behavior, and the increase in elastic recovery at higher tableting speeds for ibuprofen formulations has also been reported in the literature [49]. It is important to note that high elastic recovery can lead to a reduction in the bonding area, interparticle bonding, and lower tablet strength, while increased elastic recovery in the decompression phase has been associated with higher compression force and speed [50,51]. However, tablet defects associated with excessive relaxation, such as capping, were not observed in any of the samples tested.

### 3.4. Tablet Tensile Strength and Strain Rate Sensitivity

**Figure 4:**
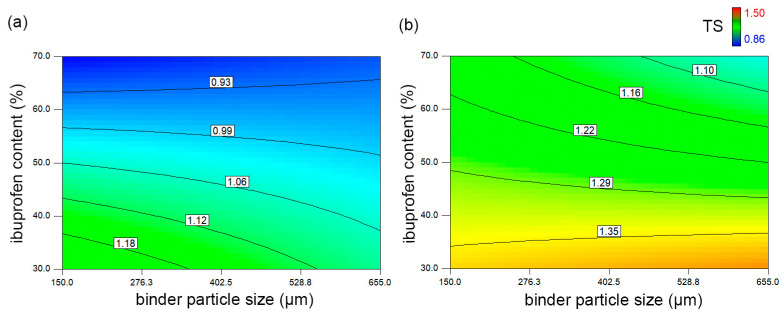
The influence of ibuprofen content and binder particle size on tensile strength at (**a**) 200 kg compression load and (**b**) 500 kg compression load.

The tensile strength values obtained for the investigated samples indicate a high dilution potential of the investigated co-processed excipients (Table 4). Considering the well-known poor compaction properties of ibuprofen and the fact that most of the tested samples, including some with 70% ibuprofen, exhibited a TS above 1 MPa, which is considered an acceptable mechanical strength, the results obtained are quite promising [44,52]. The factors that showed a statistically significant effect on tensile strength include ibuprofen content, compression load and the interaction between binder particle size, ibuprofen content, and compression load. Ibuprofen content had a negative effect on tensile strength, which was expected considering its poor compactibility. However, it can be observed that at higher compression pressure, all tablets exhibited a TS higher than 1 MPa regardless of ibuprofen content. The results also show that at higher ibuprofen content, the difference in mechanical properties of tablets prepared with two co-processed excipients becomes more prominent at higher compression loads (Figure 4). Although both co-processed excipients can provide acceptable mechanical properties at 70% ibuprofen content, a higher TS value can be achieved with C150 as its particles are smaller, and therefore a larger surface area is available for bonding between the particles.

Furthermore, the TS values of the tablets containing 70% ibuprofen and our novel co-processed excipients were comparable to the TS values reported in the literature for ibuprofen tablets containing commercially available directly compressible co-processed excipients (Ludipress^®^ and Microcelac^®^), which were prepared at even higher compression forces than those used in this study [28]. This highlights the potential of the novel co-processed excipients investigated in this study to overcome the challenges posed by the API, which is prone to elastic relaxation, resulting in poor compactibility and hence low tensile strength of the tablet prepared by direct compression.

**Figure 5:**
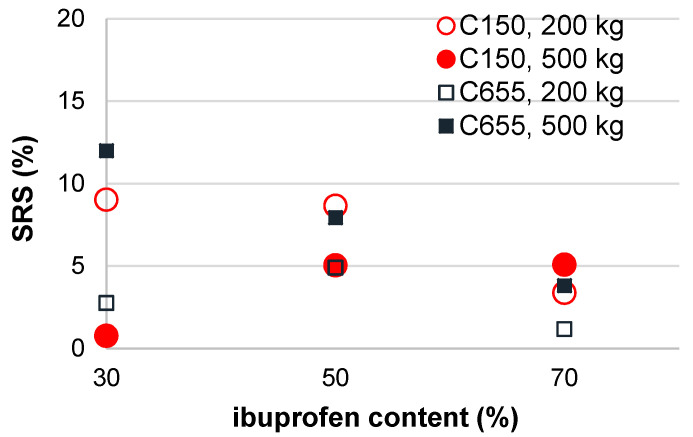
The strain rate sensitivity indices of the samples with different ibuprofen content.

It is well described in the literature that compression speed can influence the mechanical properties of tablets, with higher compression speeds being associated with a decrease in compact tensile strength. Such behavior is particularly observed in materials prone to plastic and/or elastic deformation [53]. Considering the increasing production rates of modern tableting machines, it is of utmost importance to investigate the influence of compression speed on tablet properties in order to avoid serious tablet defects that might occur during the scale-up process due to the increased production speed. Interestingly, the compression speed did not show a significant effect on the tensile strength of the tablets prepared with ibuprofen and our co-processed excipients. This is further depicted by the values of the SRS indices, which are well below 10% in the case of C150 and equal to or below 12% in the case of C655, which can be regarded as a relatively low sensitivity to changes in compression speed [54,55]. Interestingly, it can be observed that for 70% ibuprofen, the differences in SRS indices determined for two co-processed excipients at the same compression pressure are even smaller and none of the values exceeded 5% (Figure 5). This is particularly promising considering that the formulations studied contain a high content of ibuprofen, which is known to undergo both plastic and elastic deformation during compression [53]. Furthermore, the influence of compression speed on ER observed in this study and the viscoelastic properties of ibuprofen described in the literature underline the importance of the acceptable mechanical properties observed.

### 3.5. Dissolution Studies

**Figure 6:**
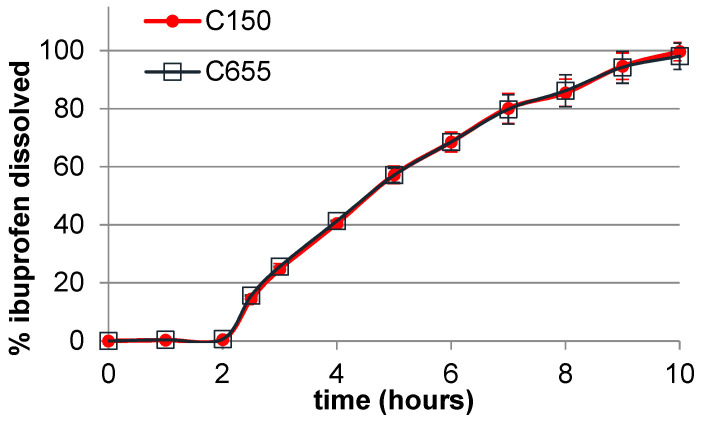
In vitro dissolution profiles of tablets containing ibuprofen (70%) and co-processed excipients (30%).

The results of dissolution studies for tablets prepared with 70% ibuprofen and two co-processed excipients are shown in Figure 6. It can be observed that the dissolution profiles of the tablets prepared with two co-processed excipients are almost superimposable. This finding indicates that the particle size of the lipid binder used to prepare the co-processed excipients, and consequently the particle size and morphology of the co-processed excipients, have no effect on the dissolution rate of ibuprofen. Thus, the results obtained show that the particle size and morphology of co-processed excipients can be tailored to achieve targeted flow and compaction properties without affecting dissolution behavior. Furthermore, these novel lipid-based co-processed excipients enabled prolonged ibuprofen release over 10 h, which indicates that a lipid matrix was formed. The results of the analysis of ibuprofen release kinetics are shown in Table 6. The highest correlation coefficient (r^2^) shows that ibuprofen release from the studied tablets follows zero-order kinetics, indicating a constant release rate of ibuprofen, which is mainly governed by the surface erosion of the lipid matrix. Among the co-processed excipients available on the market, there is a lack of directly compressible co-processed excipients for the production of modified-release tablets. In addition, there is limited research addressing the development of co-processed excipients for modified-release tablets, and the reported studies refer to either hypromellose-based excipients or lipid-based excipients, which are prepared using relatively complex, multi-step, and energy-intensive methods [21,22,23]. This emphasizes the potential of our lipid-based excipients, obtained by a simple, solvent-free process, for the production of modified-release tablets by direct compression.

## 4. Conclusions

The present study tackles the lack of directly compressible co-processed excipients for modified-release formulations. The results presented show that the co-processing of lactose monohydrate and glyceryl palmitostearate by fluidized bed melt granulation can provide a promising excipient for the direct compression of highly dosed challenging APIs such as ibuprofen. The particle size of glyceryl palmitostearate and consequently the size and shape of the co-processed excipient particles showed a pronounced influence on flowability, while the influence on compaction properties and ibuprofen dissolution was less pronounced. Although the compression parameters showed an influence on the compaction behavior of the investigated tablet blends, the influence of compression speed on tensile strength was not significant, indicating that the investigated co-processed excipients can provide good mechanical properties of tablets produced at high tableting speeds. The investigated co-processed excipients enabled direct compression of tablets with an ibuprofen content of up to 70%, avoiding the adhesion problems typical of this API. This was reflected in low detachment and ejection stresses (<1.7 MPa) and acceptable tablet mechanical properties (tensile strength > 1 MPa) even at this high ibuprofen content. These novel co-processed excipients enabled prolonged release over 10 h from the tablets containing 70% ibuprofen, demonstrating their suitability as directly compressible modified-release excipients with high dilution potential.

## Figures and Tables

**Table 1:** The real and coded values of the investigated variables.

Independent Variable	Symbol	Lower Level (−1)	Higher Level (+)
Binder particle size (µm)	X_1_	150	655
Ibuprofen content (%)	X_2_	30	70
Compression speed (mm/min)	X_3_	60	120
Compression load (kg)	X_4_	200	500

**Table 2:** Ibuprofen flowability and particle size distribution.

CI	d10 (µm)	d50 (µm)	d90 (µm)	Span
26.24 ± 0.61 ^1^	17.7 ^2^	61.5 ^2^	122.0 ^2^	1.698 ^2^

^1^ The mean value of 3 measurements; ^2^ the mean value of 4 measurements.

**Table 3:** The properties of the co-processed excipients.

Co-Processed Excipient ^1^	A ^2^	C ^3^	R ^4^	d_A_ (µm) ^5^	CI (%) ^6^
C150	1.33 ± 0.20	0.81 ± 0.05	0.77 ± 0.11	204.4 ± 49.5	8.33 ± 0.72
C655	1.14 ± 0.11	0.86 ± 0.03	0.88 ± 0.07	498.7 ± 131.7	4.17 ± 0.00

^1^ Total number of the analyzed particles was 1806 in the case of C150 (excipient prepared with a binder particle size of ≈150 µm) and 1123 in the case of C655 (excipient prepared with a binder particle size of ≈655 µm); ^2^ aspect ratio; ^3^ circularity; ^4^ roundness; ^5^ projected diameter; ^6^ Carr index.

**Table 4:** The obtained values of the response variables.

Run No.	X_1_	X_2_	X_3_	X_4_	NW ^1^ (J)	DS ^2^ (MPa)	ES ^3^ (MPa)	ER ^4^ (%)	TS ^5^ (MPa)
4	150	30	60	200	0.43	0.43	0.33	18.1	1.33
11	655	30	60	200	0.40	0.39	0.34	12.9	1.09
6	150	70	60	200	0.40	0.78	0.66	11.4	0.89
2	655	70	60	200	0.40	0.83	0.62	13.3	0.86
7	150	30	120	200	0.48	0.56	0.50	17.2	1.21
3	655	30	120	200	0.45	0.79	0.40	16.5	1.12
5	150	70	120	200	0.46	1.64	0.77	18.3	0.86
8	655	70	120	200	0.44	1.20	0.64	18.6	0.87
14	150	30	60	500	0.62	0.55	0.63	25.8	1.31
10	655	30	60	500	0.59	0.57	0.51	22.7	1.50
13	150	70	60	500	0.60	0.92	0.88	20.1	1.18
12	655	70	60	500	0.60	1.05	1.22	18.9	1.05
15	150	30	120	500	0.67	0.74	0.64	23.0	1.32
1	655	30	120	500	0.61	1.12	0.90	25.0	1.32
16	150	70	120	500	0.63	1.34	1.14	25.1	1.24
9	655	70	120	500	0.62	1.52	1.25	27.4	1.09

^1^ Network of compression; ^2^ detachment stress; ^3^ ejection stress; ^4^ elastic recovery; ^5^ tensile strength.

**Table 5:** The effects of the independent variables on the compaction-related response variables.

Factors and Interactions	NW ^1^ (R^2^ = 0.9978)		DS ^2^ (R^2^ = 0.9474)		ES ^3^ (R^2^ =0.9406)		ER ^4^ (R^2^ = 0.9210)		TS ^5^ (R^2^ = 0.9274)	
	Coefficient Estimate	*p*-Value	Coefficient Estimate	*p*-Value	Coefficient Estimate	*p*-Value	Coefficient Estimate	*p*-Value	Coefficient Estimate	*p*-Value
Intercept	0.53		0.90		0.71		19.64		1.14	
X_1_	−0.011	0.0001	0.032	0.3172	0.021	0.3949	–	–	−0.027	0.1045
X_2_	−6.25 × 10^−3^	0.0044	0.26	<0.0001	0.18	<0.0001	−0.51	0.2305	−0.14	<0.0001
X_3_	0.020	<0.0001	0.21	0.0001	0.066	0.0193	1.75	0.0011	–	–
X_4_	0.092	<0.0001	0.074	0.0376	0.18	<0.0001	3.87	<0.0001	0.11	<0.0001
X_1_X_2_	7.50 × 10^−3^	0.0015	–	–	–	–	–	–	–	–
X_1_X_3_	−3.75 × 10^−3^	0.0464	–	–	–	–	–	–	–	–
X_1_X_4_	–	–	0.057	0.0935	0.053	0.0469	–	–	–	–
X_2_X_3_	–	–	0.053	0.1133	–	–	1.47	0.0037	–	–
X_2_X_4_	–	–	–	–	0.043	0.0946	–	–	–	–
X_3_X_4_	−5.00 × 10^−3^	0.0138	–	–	–	–	–	–	–	–
X_1_X_2_X_3_	–	–	−0.067	0.0556	–	–	–	–	–	–
X_1_X_2_X_4_	–	–	–	–	–	–	–	–	−0.049	0.0090

^1^ Network of compression; ^2^ detachment stress; ^3^ ejection stress; ^4^ elastic recovery; ^5^ tensile strength.

**Table 6:** Correlation coefficients obtained for mathematical models used to determine the kinetics of drug release.

Sample	Correlation Coefficients (r^2^)			
	Zero-order	First-order	Higuchi	Korsmeyer–Peppas
C150 ^1^	0.9741	0.8380	0.8689	0.9467
C655 ^2^	0.9742	0.8432	0.8762	0.9504

^1^ tablets with excipient prepared with a binder particle size of ≈150 µm; ^2^ tablets with excipient prepared with a binder particle size of ≈655 µm.

## Data Availability

The data presented in this study are available on request from the corresponding author.
